# Visual Measurement of Suture Strain for Robotic Surgery

**DOI:** 10.1155/2011/879086

**Published:** 2011-02-24

**Authors:** John Martell, Thomas Elmer, Nachappa Gopalsami, Young Soo Park

**Affiliations:** ^1^Department of Surgery, The University of Chicago, Chicago, IL 60637, USA; ^2^Argonne National Laboratory, Argonne, IL 60439, USA

## Abstract

Minimally invasive surgical procedures offer advantages of smaller incisions, decreased hospital length of stay, and rapid postoperative recovery to the patient. Surgical robots improve access and visualization intraoperatively and have expanded the indications for minimally invasive procedures. A limitation of the DaVinci surgical robot is a lack of sensory feedback to the operative surgeon. Experienced robotic surgeons use visual interpretation of tissue and suture deformation as a surrogate for tactile feedback. A difficulty encountered during robotic surgery is maintaining adequate suture tension while tying knots or following a running anastomotic suture. Displaying suture strain in real time has potential to decrease the learning curve and improve the performance and safety of robotic surgical procedures. Conventional strain measurement methods involve installation of complex sensors on the robotic instruments. This paper presents a noninvasive video processing-based method to determine strain in surgical sutures. The method accurately calculates strain in suture by processing video from the existing surgical camera, making implementation uncomplicated. The video analysis method was developed and validated using video of suture strain standards on a servohydraulic testing system. The video-based suture strain algorithm is shown capable of measuring suture strains of 0.2% with subpixel resolution and proven reliability under various conditions.

## 1. Introduction

The use of robotics in surgery has undergone rapid growth due to the advantages it offers of small incisions, fast recovery, and low cost [1]. As a case in point, greater than 50% of radical prostatectomies for prostate cancer in the US are now being performed with robotic assistance [2]. In robotic surgery, the robot is operated in teleoperation mode, where the surgeon controls the robot manipulator arms with hand controllers. While visual feedback is the main surrogate of sensory feedback, direct force feedback to the surgeon's hand has been deemed necessary for more difficult surgical procedures [3]. 

The recent success of the DaVinci surgical robot, which has no force feedback, demonstrates that the incorporation of high resolution binocular vision allows an experienced surgeon to use visual cues as a surrogate for sensory feedback [4]. Surgeons can effectively deduce force information by visually observing the deflection of the membrane being manipulated. Robotic surgery is currently limited to operations in which the surgeon can visually compensate for sensory feedback. The development of a sensory feedback system for robotic surgery procedures will decrease the learning curve and allow more difficult operative procedures to be performed with minimal incision robotic technique. 

One of the challenges encountered during robotic surgery is maintaining suture tension. Excessive tension during knot tying may cause breakage of suture or tissue, while insufficient tension may result in knot failure. Additionally, maintaining adequate suture tension during a running anastomotic suture is difficult even for a highly skilled surgeon [5]. It has been demonstrated that haptic feedback via visual and auditory cues does decrease the time to perform suture tying with robotic systems [6]. Several investigators (e.g., Akinbiyi et al., Shimachi et al., Reiley et al.) install complex sensors in order to directly measure forces on sutures during robot-assisted surgical tasks [7–9]. However, these sensors are generally difficult to apply to robotic surgical systems, adding considerable costs, and the risk of detachment. To this end, we present a novel, non-invasive approach to calculate suture strains directly from video images routinely available during robotic surgical procedures, thereby avoiding the installation of complex sensors. 

Measuring and displaying suture strain in real time has potential to improve the performance and safety of robotic surgical operations. While conventional strain measurement methods involve complex sensor installation, this paper presents a non-invasive method based on video image processing. In the following sections, the method and algorithm are described, and the performance of the method is evaluated in terms of accuracy and adaptability for various conditions.

## 2. Methods

### 2.1. Video Processing Algorithm

**Figure 1:**
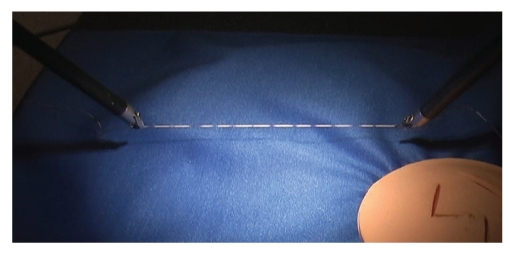
Marked suture held by the two grippers of a surgery robot.

To measure suture strain, we employ a suture that is premarked with a set of dark markers at regular intervals. Shown in Figure 1 is a marked suture held between the two grippers of a surgical robot. The suture strain is measured by video detection of the displacement of these markers upon tension. While the concept appears simple, the challenge lies in accurately determining suture strain in real time using frame-by-frame video processing to automatically identify and track markers on a suture that moves in different planes during tying. 

**Figure 2:**
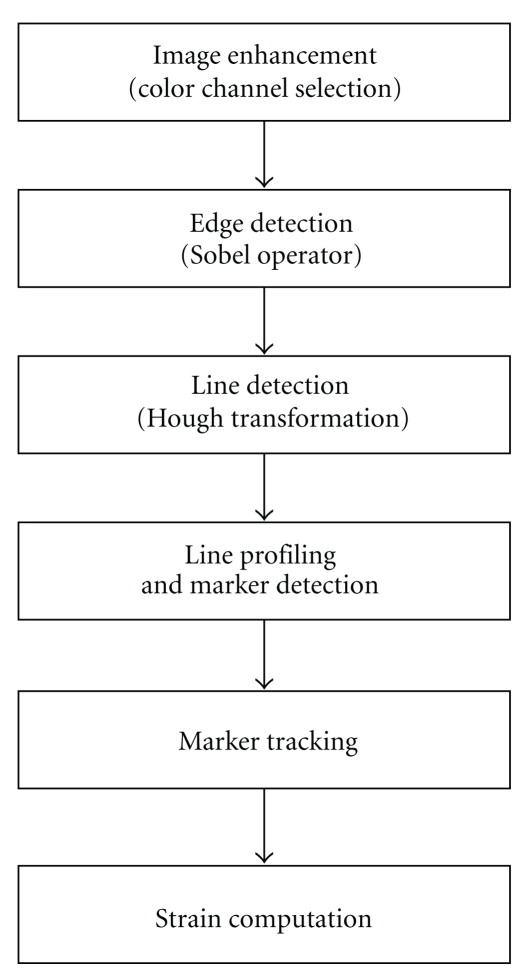
Flow chart of image processing algorithm.

The image processing algorithm is composed of the steps shown in Figure 2: image enhancement (color channel selection), edge detection, line detection (Hough transform), line profiling and marker detection, marker tracking (quadratic regression), and strain computation. Detailed description of each step is presented in this section.

#### 2.1.1. Image Enhancement (Color Channel Selection)

**Figure 3:**
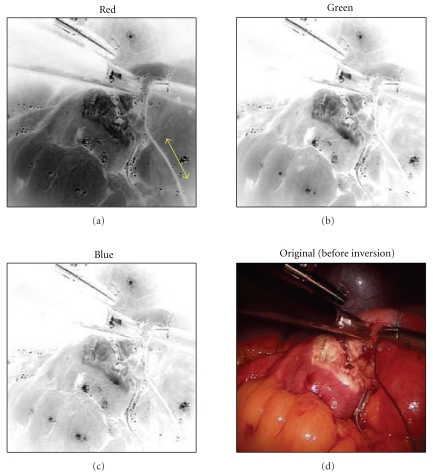
Color channel selection of dark suture (the red channel gives best contrast for suture image in the inverted image frame).

In order to allow accurate and reliable strain measurement by visual image processing, it is first important to have video image frames with good marker contrast. The algorithm can process video frames acquired from a color or B/W camera. For a color (RGB) video image, the algorithm automatically calculates and determines the color channel that gives the best contrast between background and suture. The algorithm then adapts the channel in such a way that renders the darkest background against a light suture image. For example, in the case of dark suture as illustrated in Figure 3, the grayscale image of each channel is first inverted so the suture image appears light. Then the red channel is selected for background, since blue appears light in the inverted background. 

#### 2.1.2. Edge Detection

After the color channel is selected for optimum contrast to visualize the suture, Sobel edge operators are applied to the grayscale image resulting in a binary image with enhanced suture edges for processing with the Hough transform (see Section 2.1.3). 

#### 2.1.3. Line Detection (Hough Transform)

The suture line image is identified by a line detection algorithm. Two widely known algorithms—Radon transform [11] and Hough transform [12]—may be adopted for this purpose. The Radon transform can be applied directly to a grayscale image, and thus gives flexibility. However, since it is a computationally intensive process, it is applicable to offline processing or when the suture remains relatively still. In the latter case, line detection can be performed on every *N*th frame. Every frame must be processed when the suture moves quickly. In such cases, a computationally efficient Hough transform is used, which operates only on binary images. To obtain a binary image, a Sobel edge detection algorithm is applied to a grayscale image which produces a binary (B/W) image with enhanced edges in the video frame, including the suture line.

**Figure 4:**
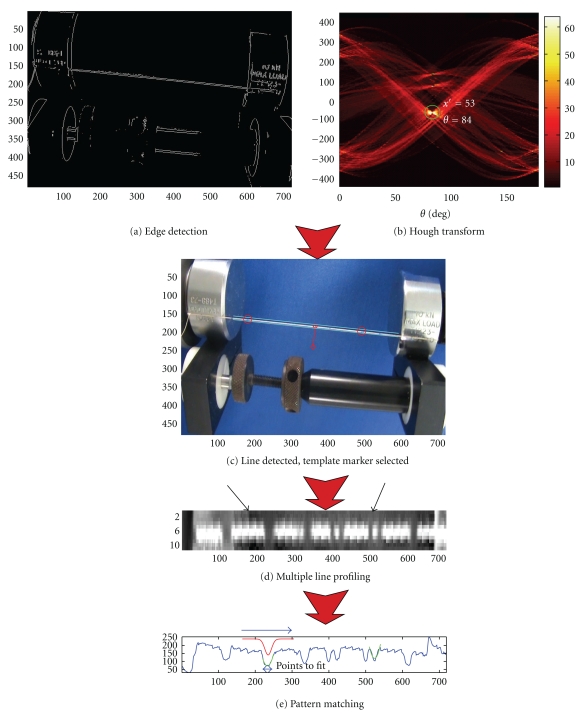
Suture line and marker detection process. (a) A binary image resulting from Sobel edge detection operators. (b) Hough transform on binary image for line detection. (c) Detected suture is displayed on the video image and markers are selected (red circles). (d) Line profile showing chosen markers (black arrows). (e) The pattern for the chosen marker (red line) is matched in the line profile (green line).

Figures 4(a)–4(c) illustrate the various steps of the automated suture line detection process.  Figure 4(a) shows the edge images obtained as a result of a Sobel edge detection algorithm. A Hough transform is then applied to the edge image to identify the suture line, which is represented by the maximum intensity point on the Hough transform. The Hough transform maps the image points (*i*, *j*) into points in a parametric space (*ρ*, *θ*) according to

(1) ρ=icos θ+jsinθ,

where *ρ* and *θ* represent the offset distance and inclination of the line from the image space origin [13, 14].  Figure 4(b) shows the results of Hough transform, where the straight suture line in the image frame is mapped to a point indicated by a circle.  Figure 4(c) shows the identified suture line displayed in the original image.

#### 2.1.4. Line Profiling and Marker Detection

The marker location is detected by intensity profiling along the suture line and pattern matching with a known “template” intensity profile representing the mark. However, because the previously detected edge line does not coincide with the suture center line, such profiling will not result in correct intensity profile. Therefore, at each incremental image point along the line, we take ±*N* lines on either side of the detected line and average them to include the center of the suture. This process of multiline profiling is illustrated in Figure 4(d), where intensity is averaged over the width of the suture line (7 pixels). This scheme also helps in cases where the “edge” line is not parallel to the suture line or the suture line becomes slack.

Along the averaged intensity profile, the location of the marker is detected by pattern matching with a known marker template. The software gives the option of manually picking the template marker points, shown as red circles in Figure 4(c), or it can be automatically picked. In this example the markers were selected manually. Once the marker point is picked, its intensity profile is used as a template throughout the tracking process. We have observed that strain values are more accurate when markers are separated by the greatest distance. Pattern matching is accomplished by computing cross-correlation of the known template and the intensity profile of the moving window, as illustrated in Figure 4(e). The correlation coefficient (*ρ*_*pq*_) between template (*p*) and suture line intensity (*q*) is defined as

(2) $$\rho_{pq}=\frac{C_{pq}}{\sigma_{p}\sigma_{q}},$$

where *C*_*pq*_ is the covariance and *σ*_*p*_^2^, *σ*_*q*_^2^ are variances of random variables *p* and *q*, respectively. As a result, the marker location along the suture line profile is estimated with a resolution of one pixel.

#### 2.1.5. Marker Tracking (Quadratic Regression)

**Figure 5:**
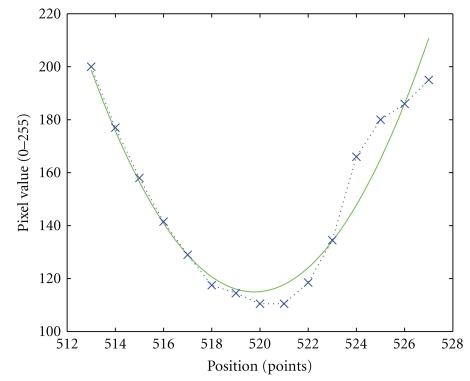
Marker tracking: quadratic curve fitting.

Once the markers are detected by pattern matching as in the previous section, the program selects a set of markers and tracks them with high precision. The markers to be tracked are selected either manually or automatically. Once the markers are chosen, the algorithm tracks them from frame to frame, and the center of the marker location is estimated by quadratic regression:

(3) $$y=ax^{2}+bx+c,$$

where *y* is the pixel intensity, *x* is the *x*-coordinate of the marker center along the line, and the constants *a*, *b*, and *c* are coefficients of the fitted quadratic curve and are determined by quadratic regression.  Figure 5 illustrates the curve fit to five discrete intensity points. The marker location is determined as the *x*-value of the minimum point of the curve. 

Determining the marker positions from a quadratic curve fit based on multiple individual pixels allows detection of the marker center position with sub-pixel resolution. The measurement resolution is expected to be dependent on the marker width and the contrast of marker edge: the greater the contrast, the larger the difference in gray level values that we have between mark and no mark, allowing positions to be measured in finer increments**. **The previous step of marker detection based on cross-correlation gives a single image pixel spatial resolution. The purpose of marker tracking is to obtain higher resolution. By adopting quadratic regression (curve fitting) on a smaller window, it will give infinite resolution of marker location.

#### 2.1.6. Strain Computation

Once the positions of the markers are known, the suture strain is calculated by

(4) $$s=\frac{\Delta L}{L},$$

where *L* is the reference suture length between markers before stretch and Δ*L* is the change in length between marker(s). Only the visible component of suture strain may be calculated. When the suture is positioned perpendicular to the video camera, the total strain and visible strain are equivalent. When the suture position is oblique to the video camera, only the component of strain perpendicular to the video camera is detectable. For the purpose of this study the suture is positioned perpendicular to the video camera. 

**Figure 6:**
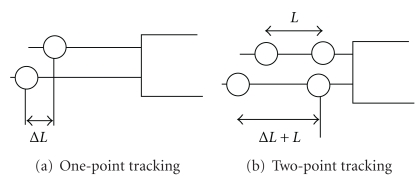
Strain methods.

Strain calculations were implemented in two ways: one-point tracking and two-point tracking. The former approach, as illustrated in Figure 6(a), assumes a fixed location of one end (absolute *L*), and the strain is solely measured by change in marker location, Δ*L*. This approach is adopted to determine the highest attainable measurement precision. In two point tracking, as illustrated in Figure 6(b), both end markers are assumed to move during stretch (relative to *L*), and the strain is computed from the relative distance between them. Implementation of this method requires development of an algorithm that can track moving suture. We expect the two-point tracking method to result in larger measurement errors compared to one-point tracking, due to additive errors in tracking two markers. 

**Figure 7:**
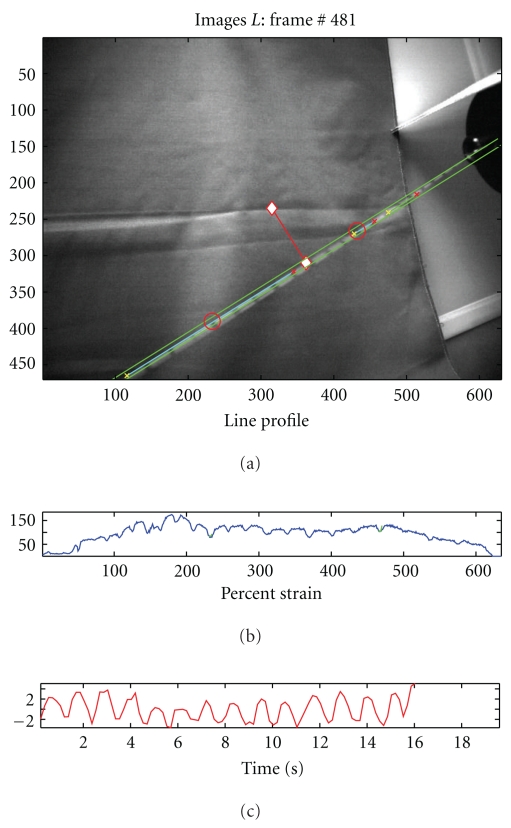
Snapshot of the video processing for strain measurement display.

Incorporating the various parts of the video processing method, the strain measurement software is constructed.  Figure 7 shows a snapshot of the video processing software which displays the video stream in the top window and the strain measurements in the bottom most plot. Shown in the middle window is the intensity profile, which is helpful for monitoring the marker detection process. When the suture is not under strain, this algorithm will yield inconsistent results. We implemented multiple line profiling to allow slackened suture to be tracked, although strain measurements are not possible under these conditions.

### 2.2. Strain Measurement Test

To evaluate the performance of the video algorithm, a series of calibrated suture loading tests were designed and performed.

#### 2.2.1. Cyclic Loading of Stationary Suture

**Figure 8:**
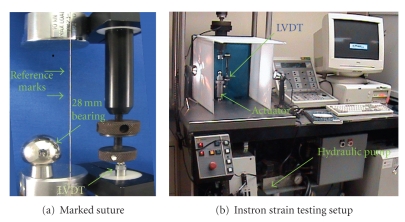
Loading test experimental setup

To evaluate the highest attainable strain resolution, a series of stationary loading tests were performed using an Instron 8500+ servo-hydraulic testing system as shown in Figure 8. 2.0 Dexon II suture material was used in the test. As shown in Figure 8(a), the suture was marked with a sharp mechanical pencil at 1 cm intervals and tied between a 500-Newton load cell and the system actuator. The suture was preloaded to 2.5 newtons, and the working length of the suture was measured. The actuator was then programmed for displacements to provide strains of 0.1%, 0.2%, 0.3%, 0.4%, and 0.45%.

**Figure 9:**
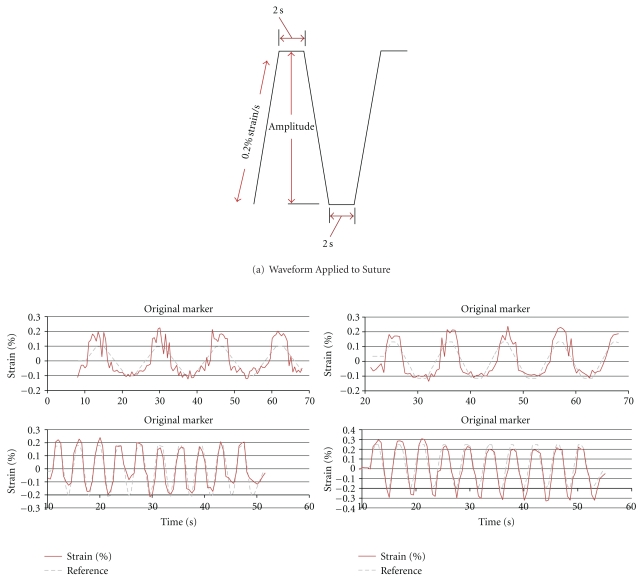
Video processing estimates of strain compared to the actual values as recorded by the materials testing system. (a) Trapezoidal reference waveform. (b) Strain measurement results.

Ten trapezoidal shaped cycles with a ramp strain rate of 0.2% strain/sec and a two-second pause at peak and trough were applied to the suture in tension (Figure 9(a)). A linear position transducer (Instron LVDT no. 2601-062, ± 0.013 mm) was used to control this portion of the testing. A digital video camera (Sony DCR-SR45, resolution: 72 × 480 pixels) was used to record the video images. The suture motion was limited to a plane perpendicular to the camera axis at a fixed distance. 

**Figure 10:**
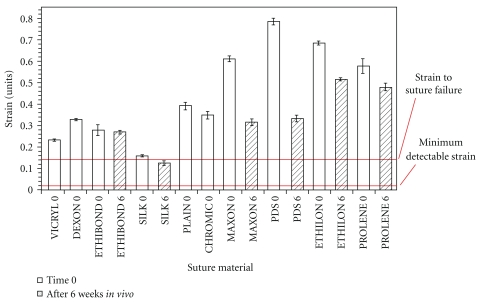
Failure strain of most common surgical sutures: Our measurement system detects a minimum of 0.2% strain in suture which is well below the strain to failure (13%) (This figure was reprinted from [10], with permission from Elsevier).

Video processing estimates of strain compared to the actual values as recorded by the materials testing system. The image processing algorithm was run offline on a recorded video stream, and the total strain amplitude was calculated based on one-point tracking methods. The calculated strain amplitudes were then compared to the actual strain amplitude recorded by the Instron testing system. The video processing algorithm was unable to detect strains in the suture below 0.2%. The amplitude and shape of the waveforms resulting from the video strain detection algorithm matched the displacements produced by the material testing system as strain increased beyond 0.2%.  Figure 9(b) shows improved fit of the displacement waveforms calculated with the video system to the actual displacements applied to the suture by the testing system. The accuracy of the video algorithm is estimated as the calculated strain compared to the magnitude of strain applied by the testing system. The majority of commonly used surgical sutures have been reported to fail at above 20% strain (i.e., >0.2 strain as shown in Figure 10). In comparison, the algorithm sensitivity was capable of detecting suture strain with high measurement resolution at strain values well below the suture failure strain of 20% (0.2 strain). 

#### 2.2.2. Effects of Sharper Marker

**Figure 11:**
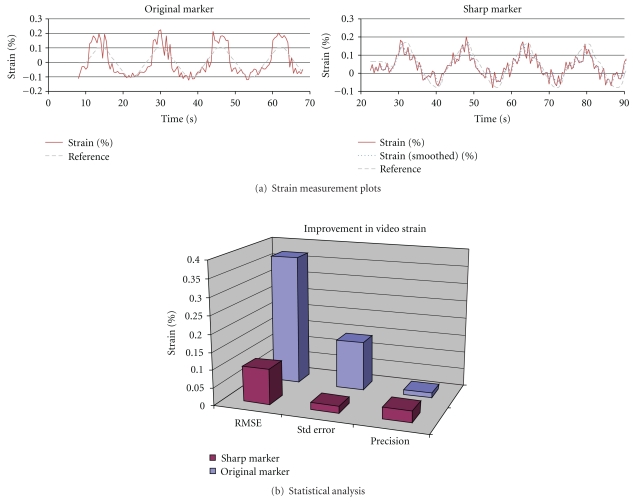
Comparison of strain measurement for different contrast markers for 0.2% strain loading cycle.

We expected the shape and contrast of the markers to impact the performance of this visually based strain detection algorithm. Specifically, the marker tracking algorithm applies curve fitting to the marker edge based on intensity variation. Accordingly, it was expected that markers with sharper (higher contrast) edges would allow for superior measurement resolution. To investigate such an effect, specimens with different contrast markers were tested as in the previous section. The video algorithm's strain measurement of each marked suture was compared to its corresponding measurement as recorded on the Instron testing system.  Figure 11 shows the strain measurement result for a loading cycle of 0.2% strain. In the plot, the strain measurement with sharper marker (top plot) is placed in comparison with the previous measurement on a less sharp marker (bottom plot). The sharp marker resulted in more accurate measurement (standard deviation (std) = 0.04; root mean square (RMSE) = 0.04) compared to the previous results (std = 0.06 and RMSE = 0.06).

#### 2.2.3. Two-Point Marker Tracking

**Figure 12:**
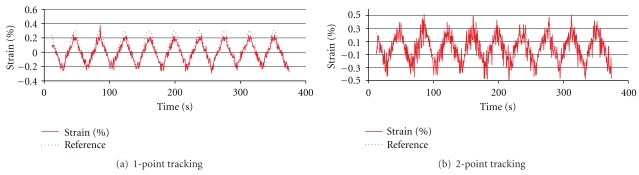
Comparison of measurement results with one-point and two-point tracking.

The previous stationary loading tests calculated strain using a single marker and assumed that the other end of the suture was fixed; therefore, the video images were processed with one-point marker tracking. While its purpose was to determine the maximum attainable measurement resolution, both end markers may move significantly from frame to frame in clinical situations. In this regard, we implemented an improved algorithm that selects an appropriate set of markers, tracks them, and computes strain under dynamic conditions. Tensile testing loads were applied to marked sutures as described above, with postprocessing performed using one- and two-point tracking, allowing an assessment of the precision of marker tracking methods.  Figure 12 shows that the accuracy of strain measurements for two-point tracking (RMSE = 0.11), was lower than that for one-point tracking (RMSE = 0.06). The smallest detectable strains for one- and two-point tracking were 0.2% and 0.5% strain, respectively. 

## 3. Conclusion

We have introduced a video processing method that detects suture strain during robotic surgery. This video-based method employs a suite of image processing algorithms that identify the suture line, locate and track the displacement of markers on the suture, and convert marker displacements into suture strain values. The method is capable of tracking markers on a moving suture, which is necessary in surgical procedures. The performance of this video method was evaluated by a series of strain tests using a hydraulic testing machine and 2.0 Dexon II suture. These tests showed a minimum detectable strain of 0.2% for one-marker tracking on stationary suture and 0.5% for the more clinically relevant two-marker tracking on moving suture. These minimum detectable strains are two orders of magnitude smaller than the known strain to failure of most suture materials (20+%), allowing a large margin of safety in the clinical setting. 

Further improvement in the resolution of this technique is expected with commercially applied high contrast marked suture. A limitation of the current video processing algorithm is the inability to accurately detect strain when the suture lies at an oblique angle to the video camera. We are currently developing a stereoscopic imaging algorithm that calculates the angle between the suture and the video camera. Given this information, the visible suture strain is used to determine the total suture strain. The addition of the stereoscopic imaging will make the clinical application more robust. Real-time feedback of suture tension during robotic-assisted procedures is expected to compensate for the current lack of sensory feedback in robotic surgery, thereby decreasing the learning curve and improving the safety of robotic-assisted procedures.
